# Integrating Connected Vehicles into IoT Ecosystems: A Comparative Study of Low-Power, Long-Range Communication Technologies

**DOI:** 10.3390/s24237607

**Published:** 2024-11-28

**Authors:** Valentin Iordache, Marius Minea, Răzvan Andrei Gheorghiu, Florin Bădău, Angel Ciprian Cormoș, Valentin Alexandru Stan, Ion Nicolae Stăncel, Victor Stoica

**Affiliations:** Transport Faculty, National University of Science and Technology POLITEHNICA Bucharest, 060042 Bucharest, Romania; marius.minea@upb.ro (M.M.); andrei.gheorghiu@upb.ro (R.A.G.); florin.badau@upb.ro (F.B.); angel.cormos@upb.ro (A.C.C.); valentin.stan@upb.ro (V.A.S.); ion.stancel@upb.ro (I.N.S.); victorstoica114@gmail.com (V.S.)

**Keywords:** V2I, V2IoT, Bluetooth, ZigBee, LoRa, nRF24, long-range, vehicle

## Abstract

Integrating road vehicles into broader Internet of Things (IoT) ecosystems is an important step in the development of fully connected and smart transportation systems. This research explores the potential of using communication technologies that achieve a balance between low-power and long-range (LPLR) capabilities while remaining cost-effective, specifically Bluetooth Classic BR-EDR, Bluetooth LE, ZigBee, nRF24, and LoRa—for Vehicle-to-Infrastructure (V2I) and Vehicle-to-IoT (V2IoT) ecosystem interactions. During this research, several field tests were conducted employing different types of communication modules, across three distinct environments: an open-field inter-urban road, a forest inter-urban road, and an urban road. The modules were evaluated based on the communication range, messaging rate, error rate, and geographical data from GNSS (Global Navigation Satellite System) coordinates, using point-to-point communication between a roadside unit (RSU) and a moving vehicle equipped with an onboard unit (OBU). The results demonstrate the usability of these technologies for integrating vehicles into both public infrastructure (for V2I services) and private IoT systems, highlighting their potential for scalable, cost-effective deployment in smart transportation systems.

## 1. Introduction

V2IoT (Vehicle-to-Internet of Things) refers to the concept of connecting vehicles to an IoT network, allowing them to communicate and interact with other devices or systems, and enabling a wide range of applications and services. In an Internet of Things (IoT) network, vehicles are considered nodes capable of collecting, processing, and transmitting data. Communication technology is the backbone of any V2IoT solution, as it enables the seamless exchange of data between vehicles and the surrounding infrastructure, devices, and networks. V2IoT was mentioned in the Autopilot project, which focused on integrating automated vehicles into IoT systems in smart cities, allowing them to act as mobile sensors and enabling access to a wider volume of data [1]. With IoT connectivity enabled, vehicles can access essential information, helping to overcome the limitations and sometimes lack of data from their own sensors, for example, by providing navigation functions in areas without GNSS (Global Navigation Satellite System) signals, thus making driving safer and more efficient. This integration also facilitates improved traffic management through dynamic traffic light algorithms and real-time communication between vehicles and infrastructure, helping to reduce congestion and emissions [2]. V2IoT enables vehicles to access critical information beyond their immediate environment, enhancing road safety and accident prevention, particularly in autonomous driving scenarios [3]. Additionally, the use of Intelligent vehicle agents (IVAs) in urban areas allows for effective management of traffic data, ensuring fast responses to traffic conditions and minimizing delays in communication [4]. Infrastructure costs could also be diminished by employing “floating cars” (vehicles equipped with devices that collect and transmit real-time data) as moving sensors in traffic, used to collect information regarding traffic flows, congestions, accidents, weather conditions, etc.

Agent-based transportation systems enable distributed subsystems, as part of broader intelligent transport systems, to collaborate in managing and controlling traffic, based on real-time conditions. The communication network interconnecting their subsystems is of real importance for ensuring seamless, coordinated, and safe operation of all elements. A multi-agent system (MAS) is used for modeling and simulating traffic systems, as it provides an intuitive framework for representing each autonomous entity at an individual level. The MAS has been extensively applied in areas such as collaborative driving, agent-based driver behavior modeling, and urban traffic management and control (UTMC) [5,6].

### 1.1. Integration of Vehicles into Private IoT Ecosystems, Enterprise Fleet Management, or Smart Home Systems

**Figure 1:**
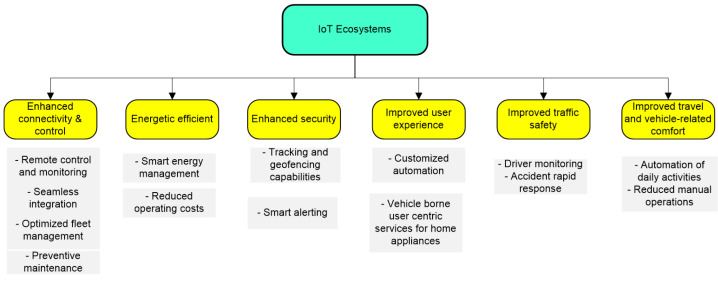
Taxonomy of the benefits that integration of vehicles in IoT ecosystems can provide.

The integration of automobiles into IoT networks offers many advantages. Such ecosystems facilitate information exchange, enhancing the traffic experience, comfort, safety, and security for vehicles and their owners, even beyond driving or vehicular applications. Figure 1 shows some of these benefits.

Improved connectivity brings many advantages in terms of integrating and harmonizing information from/to different sources/destinations: remote control and monitoring of vehicles (especially for fleet owners), or enterprise/home appliances for vehicle drivers. Vehicle monitoring systems (typically known as automatic vehicle monitoring, or AVM) can be fully integrated into such an ecosystem. Functions such as the fuel level, battery status, location tracking, tire pressure, freight condition, alarm status, remote maintenance monitoring, and security alarms are just some of the features that could benefit from this integration. On the other hand, vehicles could be enabled with remote control of garage door operations, barriers, automatic e-payment, and/or home appliances’ operation at the vehicle approach.

Energy is another area where the integration in IoT environments could be beneficial, including smart (vehicle grid) management, energy consumption monitoring and/or switching loads, home appliances’ remote control, and so on. Similarly, security can be improved by remote alerting when suspicious activity is detected. Smart home systems can also be integrated into the ecosystem by activating cameras or alarms. Geofencing and tracking are other features that are important: via tracking and/or mapping of vehicles, geofencing can be set up to alert users if a vehicle leaves a designated area.

Another interesting benefit of this integration is the possibility to remotely access different states of both vehicles and their drivers, including health features related to traffic safety. When an unexpected accident occurs, the integration in the IoT ecosystem could also help in rapid responses, such as remote safety (vehicle speed at impact, position after impact, and number of passengers), or medical (state of the driver before impact, health problems, etc.) information collection.

Last, but not least, IoT-enabled vehicles could serve as moving sensors, helping to reduce the amount of equipment installed in the infrastructure, energy consumption, and maintenance operations—all these contributing to a more friendly impact over the environment of the road transportation systems.

### 1.2. Analysis of Possible Challenges

Traditional Vehicle-to-Infrastructure communications, also known as V2I (Vehicle-to-Infrastructure), have some limitations, compared to using traditional low-power, low-cost communication solutions:

- Limited interoperability, due to the less developed standardization and expansion of these solutions around the world. Also, the employment of different communication technologies and protocols could lead to more fragmentation of the services allowed, making it more difficult for vehicles traveling in different countries to access similar services, and/or maintaining seamless V2I communication.
- Since infrastructure-embedded sensors and equipment are mainly used, operation and maintenance costs are higher compared to an integrated vehicular IoT ecosystem.
- Scalability and inter-connectivity between different subsystems are dependent on standardization, and this can be challenging due to the scale and diversity of the road networks.
- Concerns are also related to the latency of messaging and bandwidth limitations, especially for older standard technologies.
- Cybersecurity is also something that should not be overlooked. Older V2I communication standards might be prone to hacking, spoofing, or data interception, potentially compromising vehicle and/or infrastructure security.
- In countries with traditional communication infrastructure, which is not fully adapted to high-speed data transmissions, applications may also suffer from latency or integration issues.
- Local, national, and international laws and standards’ diversity can lead to discontinuities in data access, while in an integrated IoT environment, using Internet addressing can help mitigate this issue and ensure seamless application functionality.
- Energy consumption of a specific set of IoT-enabled devices might be easier managed than those in a traditional V2I network. Newly developed IoT smart grids can provide intelligent monitoring, such as smart metering and sensing, predictive analytics, smart distribution of energy and consumption management, self-configuration of the grid based on load balancing, smart storage and storage management, and an improved resilience to disasters. All these, included in a transport network, bring significant benefits, such as enhanced power-supplying stability, reduced carbon emissions, cost savings, and better interconnectivity with the national energy distribution grid.

### 1.3. Benefits of Introducing LPLR Communication Modules in a Vehicular IoT-Enabled Environment, as Viable Alternatives

**Figure 2:**
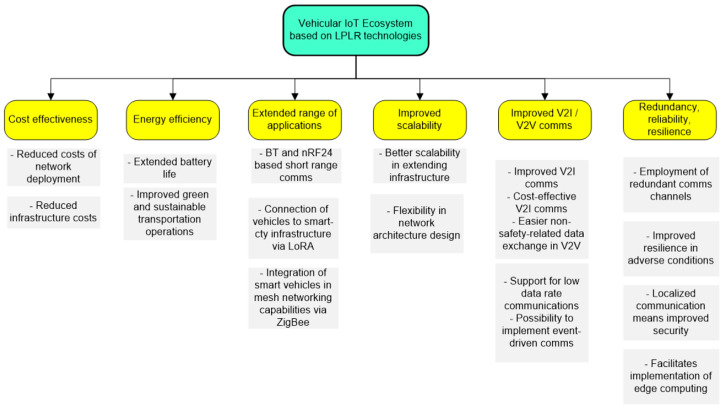
Taxonomy of estimated benefits that result from the introduction of LPLR and low-cost communication technologies in a vehicular IoT environment.

The present research is focused on the assessment of the benefits that LPLR (low-power, long-range) and low-cost communication technologies, such as Bluetooth, ZigBee, nRF24, or LoRa, could bring in a vehicular IoT environment. As a preliminary analysis showed, based on the scientific literature [7,8,9] there are several possible benefits of this enterprise, as shown in Figure 2.

Some of the most significant advantages include enhanced security, energy efficiency, flexibility, scalability, and low costs. However, as this field is relatively new, further research and integration for communications are needed before a coherent standardization will allow for harmonization of all these services. It is also recommended that this process follows a step-by-step strategy, ensuring compatibility with adjacent standards and older communication protocol versions.

While many LPLR and low-cost communication technologies are characterized by lower data rates, they are highly efficient for transmitting essential, compact data that support various V2IoT applications. These technologies prioritize power efficiency and range, making them well suited for the transfer of small, critical data packets. Examples of data and information types that can be effectively exchanged are as follow:

- In smart parking applications, data about the status of parking spaces can be sent to guide vehicles to available spots and update parking management systems, time data that will show how long a vehicle has occupied a space, or basic vehicle ID or license plate information that can be used for spot reservation and monitoring.
- Data indicating whether a vehicle has permission to enter restricted areas can be sent to be used in access control applications, along with gate control commands and vehicle/driver identification data.
- For vehicle diagnostics purposes, the fuel level, battery status, tire pressure, engine temperature, and other indicators can be transmitted periodically (for remote monitoring) or as alerts (when maintenance is needed).
- Vehicle dynamics and environmental monitoring can be accomplished by sending data about location (e.g., GPS coordinates), speed, and environmental readings (e.g., temperature, humidity, and air quality).
- Incident and safety alerts can be sent from the infrastructure to the drivers, such as congestion warnings, road hazard alerts, or weather-related warnings that are usually event-driven.
- In traffic light systems, real-time data on the current light phase can be sent to inform approaching vehicles, enabling better speed adjustments, or information from authorized vehicles (e.g., emergency vehicles and public transport) can be received to trigger priority green lights.

**Figure 3:**
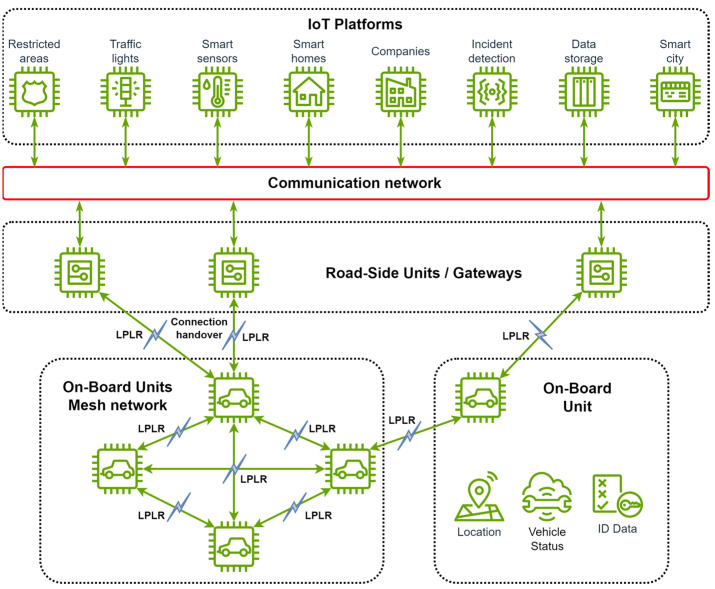
Typical elements and communication processes in a V2IoT system.

The diagram presented in Figure 3 illustrates the key elements of a V2IoT system, highlighting the communication process and deployment of LPLR communication technologies.

To evaluate and identify the best-suited communication technologies for V2IoT applications, the present research focuses on understanding each technology’s performance under varying conditions. Based on this goal, the objectives of the present research are as follow:

- To assess the capabilities of current LPLR and low-cost communication technologies, which are not necessarily developed for the automotive industry, but could be used, due to their mentioned advantages, as part of the communication infrastructure in a vehicular IoT environment.
- To evaluate the performance of LPLR and low-cost communication technologies in different scenarios and propagation conditions, including peri-urban or rural, wooded areas, and urban areas.
- To evaluate the feasibility and performance of various communication modules in enabling V2I and V2IoT interactions, with a focus on their integration into the broader IoT ecosystem.
- To highlight how LPLR low-cost communication modules, such as Bluetooth, ZigBee, nRF24, and LoRa, might serve as viable alternatives or complements to more traditional (and more expensive) V2I communication technologies.

## 2. Related Work

The focus on intelligent transportation systems is well known today due to their significance in various areas: reducing the carbon emissions and footprint of transportation, innovating solutions for safer road traffic, improving average traffic flow, and increasing the comfort of travel. All these improvements are made, in a significant amount, using information technology, artificial intelligence, and intelligent agents. Industry, academia, and the public sector participated in a series of meetings and seminars on Sustainability for Transportation and Logistics (DHW-STL), Distributed/Decentralized Hybrid Symposia on Sustainability for Transportation and Logistics (DHS-STL), and Distributed/Decentralized Hybrid Conferences on Sustainability for Transportation and Logistics (DHC-STL), to design potential solutions, possibilities, and perspectives driven by intelligent vehicle (IV) technologies to achieve sustainable and reliable intelligent functional components for traffic, transportation, and/or logistics [10]. These meetings revealed that certain embedded technologies, such as cooperative collision avoidance systems (CCAS), connected and autonomous vehicles (CAV), and advanced driver assistance systems (ADAS), are important structural and functional elements that significantly enhance traffic safety, reduce greenhouse gas emissions, and improve traffic flow [11,12].

However, telecommunication solutions have also been extensively researched, as they are a critical component of connected and intelligent vehicles. There are many obstacles to overcome, particularly since the use of radio waves for propagation entails several concerns, including environmental variability, propagation problems, interference, unauthorized information access, and channel flooding. Given the scenarios that could arise from field testing and real-world implementation, the list of issues could become significantly more complex.

Therefore, we emphasize the importance of preliminary studies on mobile communication solutions in diverse scenarios to better understand radio signal behavior across various environments. While there is an expanding body of literature on cooperative diversity, much of the current research related to communications for vehicular cooperative applications is primarily focused on the Rayleigh fading channel model. This model typically assumes a wireless communication scenario involving a stationary base station antenna positioned above the rooftop level and a mobile station located at the street level. However, some works tried another approach, i.e., cascaded Nakagami fading, such as in [13], where the authors considered that such a channel model offers a more realistic representation of an intervehicle communication scenario, where two or more independent Nakagami fading processes are assumed to result from separate groups of scatterers surrounding the two mobile terminals. Similarly, Zhou et al. found that due to the open and diverse nature of IoT channels, sensitive information becomes vulnerable to unauthorized access, leading to potential network interruptions and significant security concerns. This vulnerability restricts the use of IoT for transmitting sensitive data. To limit these vulnerabilities, they proposed a multi-antenna secure communication model based on decoding forwarding (DF) relay [14]. They considered that this solution is more suitable for the nonlinearity of IoT secrecy data.

Another direction of research has been pointing toward the usability of communication technology, in terms of delays. It is a well-known fact that some categories of messages in vehicular communication, such as EWS (emergency warning signing), are required to be delivered to the recipient in the fastest possible mode. Shao et al. [15] proposed a real-time streaming anomaly detection method based on a Bloom filter combined with hashing, with the aim of identifying anomalies in the simulation data streams, to finally provide an accurate data flow detection and ensure a proper, secure data transfer. Similar research was conducted by Alzubi [16]. This author proposed a blockchain-assisted data-securing system for medical IoT devices using the Lamport Merkle Digital Signature (LMDS). While its application is in the medical area, the solution could also be employed in vehicular communications.

The integration of vehicles into private IoT ecosystems has the potential to enhance operational efficiency, safety, and user experience. However, this integration also presents several technical challenges related to monitoring, data management, security, and interoperability. The monitoring of vehicle parameters is made possible using communication networks and sensors, which are integrated into IoT-based systems. These data are then transmitted to the cloud for detailed analytics, thereby increasing reliability [17]. Master data management in these ecosystems optimizes accuracy and reduces the data integration time, which is essential for managing the huge volume of data generated by vehicles [18]. At the same time, security is a significant problem, especially considering the challenges and characteristics specific to the implementation of secure and robust systems for IoT devices [19], and a proposed authentication scheme ensures secure communication between vehicles and IoT devices while preserving privacy [20].

## 3. Communication Technologies’ Overview

A useful methodology for selecting a communication technology tailored to V2IoT applications involves evaluating a range of appropriate existing communication solutions across various scenarios. This approach allows for assessing and analyzing their performance metrics to determine their efficacy and suitability for intended use cases. An important factor for selecting communication technologies for V2IoT applications is the effective communication range.

Originally designed for building PANs (Personal Area Networks), Bluetooth is presented in numerous papers [21,22,23,24] as an attractive solution due to its relatively low cost, energy efficiency, availability, and suitability for V2I communications [25]. Considering the future implementation of IoT internal sensors in vehicles, Mirza et al. [26] proposed a solution consisting of a low-cost and energy-efficient communication between sensor nodes and the ECU (Electronic Control Unit) employing BLE (Bluetooth Low Energy) within the vehicular ad hoc network, for an intra-vehicle wireless sensor network.

The range of Bluetooth communication has expanded with its various versions. Bluetooth 1.0 through 3.0, commonly referred to as Classic Bluetooth, support communication over distances ranging from 10 to 30 m, depending on the device class. Bluetooth 4.0 (BLE) extended the effective communication range to 100 m, while mainly focusing on improving energy efficiency [27,28].

Bluetooth 5.0 and upwards improved the communication range massively, with distances up to 300 m being achieved in VANET-type (vehicular ad hoc network) applications [28,29]. The 5.1 version increased accuracy for both indoor and outdoor applications by making use of Angle of Arrival (AoA) features [30].

ZigBee is also a very promising solution for vehicular communications, as shown by the research in this direction. A focus on IEEE 802.15.4 standard implementation in automobiles for data communication, or among the vehicles, was provided by Lakshmi Shree et al. [9]. The authors of this paper proposed the design and implementation of a ZigBee Area Network (ZAN) for VANET applications. Using IoT in vehicles could prove beneficial in ensuring preventive maintenance and better traffic safety. Similar research may be found in [31,32].

For indoor applications, ZigBee communication is limited to an average of 30 m. The effective communication range is highly influenced by factors such as physical obstacles and disturbances generated by other communication technologies occupying the same bandwidth [33,34]. Outdoor range can extend up to 600 m depending on the antenna type that is used [35].

Another communication technology that is worth attention for this type of application is nRF24. A comprehensive study regarding comparative criteria for different types of communication technologies for a vehicular environment was provided by Yogarayan et al. in [7]. The authors explored three different technologies for vehicular communications, and a direction to develop a conceptual approach to V2V (Vehicle-to-Vehicle) communication with HC-12, nRF24L01, and XBee. The HC-12 is a reliable semi-duplex wireless communication mode that provides a frequency spectrum of 433.4 to 473.0 MHz. The authors found that HC-12 and XBee work over wider distances than nRF24L01.

As with the previously outlined technologies, nRF24 communication parameters are strongly influenced by environmental factors. In various outdoor testing scenarios, communication employing nRF24 was viable up to 1900 m [36].

Especially designed for long-range applications, LoRa offers the largest range among the considered technologies. The typical range for communication is limited to around 3 km for cities but can extend to 133 km in the case of the 2.4 GHz version [37]. The large area that can be covered by LoRa devices makes it the prime solution for agricultural IoT systems, vehicle communications, and smart city applications [38].

To conclude, it appears that the evaluation of LPLR communication technologies is insufficiently explored in the scientific literature, presenting a valuable opportunity for this research. LPLR technologies have been assessed in terms of performance, especially in different fields of activity than vehicular applications; however, as our analysis suggests, they prove to be an attractive solution in the development of an IoT-based vehicular environment, primarily due to their reduced costs, scalability, maintenance, and energy efficiency.

Similar research considering comparisons between these technologies can be found in [7,32,39,40,41,42,43].

## 4. Experimental Setup

### 4.1. Test Environment

In this research, three distinct environments, grouped into two types of roads, were selected to evaluate the performance of the wireless communication modules in real-world scenarios. The selected environments represent typical use cases where V2IoT communication systems are expected to operate and are described as follows:

- An urban environment, also considered as an urban road type.
- An open-field environment, also considered as an inter-urban road type.
- A forest environment, also considered as an inter-urban road type.

**Figure 4:**
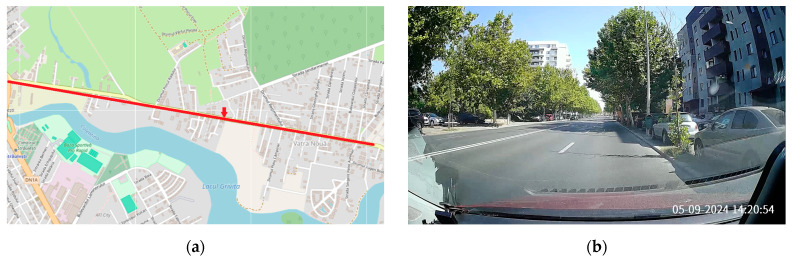
The urban environment: (**a**) Representation on a map, with the red line marking the selected road and the red arrow indicating the position of the roadside unit (source: © OpenStreetMap contributors (“https://www.openstreetmap.org/copyright” (accessed on 29 September 2024))). Map data are available under the Open Database License (ODbL). (**b**) Photo.

The urban environment is characterized by high-density infrastructure, including buildings, streets, high traffic density, and other obstacles that may interfere with wireless signals. In this environment, communication modules encounter significant challenges due to signal reflection, multipath propagation, and interference from various electronic devices. The selected road had 4 lanes (Figure 4, with the red arrow indicating the position of the roadside unit (RSU)), 2 in each direction, 2.8 km in length, and a traffic density of about 2400 vehicles per hour (both directions). The surrounding area consisted mainly of two-story houses, with some taller buildings reaching up to ten stories. Trees lined much of the street, sidewalks were often occupied by parked vehicles, there were few pedestrian crossings, and there were traffic lights at both ends of the road. The weather conditions were clear, with temperatures around 28 degrees Celsius.

**Figure 5:**
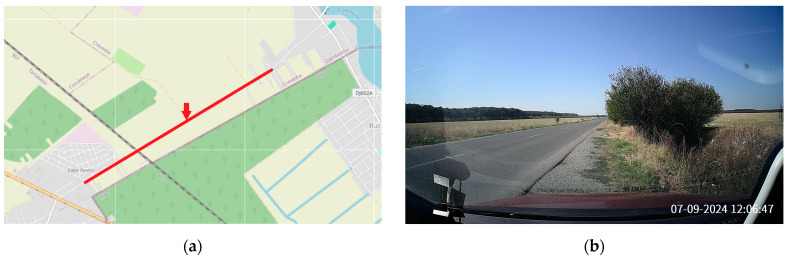
The open-field environment: (**a**) Representation on a map, with the red line marking the selected road and the red arrow indicating the position of the roadside unit (source: © OpenStreetMap contributors (“https://www.openstreetmap.org/copyright” (accessed on 29 September 2024))). Map data are available under the Open Database License (ODbL). (**b**) Photo.

The open-field environment represents rural or inter-urban areas with fewer physical obstructions, wide-open spaces, and a longer line-of-sight for signal propagation. In this environment, the primary challenge is maintaining long-range communication without the benefit of nearby infrastructure to boost or relay signals. The selected road had 2 lanes (Figure 5), 1 in each direction, 3 km in length on a flat terrain, and a traffic density of about 400 vehicles per hour. The surrounding area consisted of minimal vegetation, and few one- or two-story houses at both ends of the road. The weather conditions were clear, with temperatures around 30 degrees Celsius. Although there was a railway crossing the road, train traffic was minimal, and the measurements were paused whenever trains passed.

**Figure 6:**
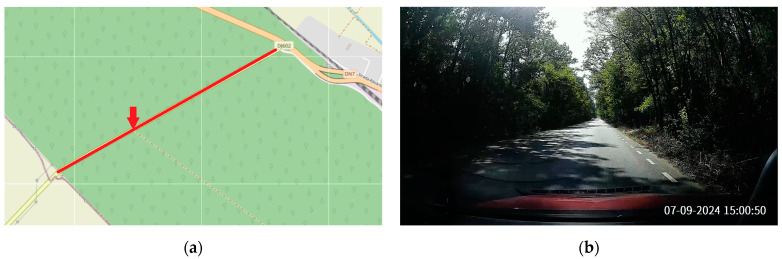
The forest environment: (**a**) Representation on a map, with the red line marking the selected road and the red arrow indicating the position of the roadside unit (source: © OpenStreetMap contributors (“https://www.openstreetmap.org/copyright” (accessed on 29 September 2024))). Map data are available under the Open Database License (ODbL). (**b**) Photo.

The forest environment represents a scenario where natural obstructions, such as trees and dense foliage, significantly impact wireless communications. Forest roads are typically narrower and more challenging for signal transmission due to absorption and scattering caused by tree leaves and other vegetation. Unlike the urban environment, the forest road lacks man-made infrastructure that could aid in signal relaying, and the conditions here create a challenging test for range and reliability. The selected road had 2 lanes (Figure 6), 1 in each direction, 1.8 km in length on a flat terrain, and a traffic density of about 600 vehicles per hour. The surrounding area consisted of tall trees and ground vegetation, with a high percentage of the road covered by tree canopy. The weather conditions were clear, with temperatures around 27 degrees Celsius.

### 4.2. Hardware Configuration

For mobile applications, the process of selecting proper communication modules is essential to ensure effective data exchange between vehicles and the road infrastructure. To ensure the modules meet the specific requirements of the selected test environments and future implementations, several criteria were considered:

- Communication range was the key aspect of this research. Since vehicles are in motion and the time available for sending messages is limited, selecting long-range communication modules with the highest available transmission power (e.g., Class 1 Bluetooth modems, or XBee Pro modules) had the highest probability of being successful.
- Protocol support was also important, because the modules should allow integration with both older or newer IoT solutions (e.g., different versions of Bluetooth Classic, or BLE, and versions Pro or 3.0 for ZigBee). Different protocols offer different advantages in terms of range, power efficiency, and data handling.
- Cost and market availability were also considered to gain an advantage over traditional and dedicated V2V or V2I communication technologies.
- Power consumption should be low, as vehicles rely on a battery (especially when not in use). All selected communication modules have a low-power mode.
- Compatibility with microcontrollers (e.g., interfaces such as UART—Universal Asynchronous Receiver-Transmitter, or SPI—Serial Peripheral Interface) as well as ease of use and integration were important, because using modules with well-documented libraries and high community support can significantly reduce the development time and complexity.

**Figure 7:**
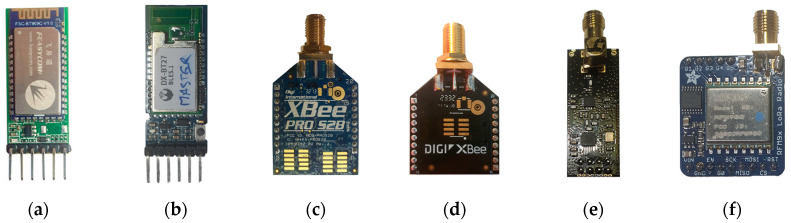
Communication modules: (**a**) FSC-BT909C, (**b**) DX-BT27, (**c**) XBee Pro S2B, (**d**) XBee 3 Pro, (**e**) nRF24L01+PA+LNA, and (**f**) Adafruit RFM95W.

Six modules were chosen for this research (Figure 7), and this section provides a brief overview of each along with key settings that were applied to them, and a comparison, focusing on their relevant specifications.

The FSC-BT909C Bluetooth module, manufactured by Feasycom (Shenzhen, China), offers a balance of compact design, energy efficiency, and affordability (it is priced at around 15 euros), simplifying the integration of wireless functionality into various products [44]. In this research, the module operates using the Bluetooth Classic BR-EDR protocol, with one unit configured as Master and the other as Slave, featuring an auto-connect function for seamless communication.

The DX-BT27 Bluetooth module, manufactured by DX-SMART Technology (Shenzhen, China), is equipped with a Bluetooth chipset and a power amplifier, offering enhanced range and high receiving sensitivity, and is compliant with the Bluetooth BLE 5.1 standard [45]. This module operates exclusively in BLE mode and, for this research, was configured in Transparent Transmission Mode. Two modules were used in a peripheral–central configuration. Priced at approximately 5 euros, it provides an affordable yet effective solution for long-range, low-power wireless communication.

The XBee Pro S2B [46] is a ZigBee communication module manufactured by Digi International (Hopkins, MN, USA) that was used to create a point-to-point network, where one node was configured as the Coordinator, responsible for managing the network, while the other served as the End-Device, communicating directly with the Coordinator. Despite being a legacy device, it can still be purchased at a price of approximately 40 euros, allowing integration with older low-power, low-data-rate applications, even when mesh networking capabilities are needed.

We also used a newer generation ZigBee device, the XBee 3 Pro. Manufactured by the same company, Digi International, similar to its predecessor, the XBee 3 Pro offers a significant communication range of up to several kilometers in optimal conditions [47]. This makes it an ideal choice for LPLR wireless networks, although it comes at a slightly higher price of around 70 euros. The same type of network was used in this research: Coordinator—End-Device.

The nRF24L01+PA+LNA module uses a Nordic Semiconductor radio transceiver [48] running a proprietary protocol, and it is manufactured by various third-party vendors in Shenzhen, China, who integrate a power amplifier (PA) and low-noise amplifier (LNA), enhancing both the transmission range and receiving sensitivity. It has a low cost, starting from around 5 euros. Considering that for this research we wanted to achieve the maximum possible range, the modules were configured to create a point-to-point network with the lowest data rate setting, maximum PA gain, and the highest permissible channel according to regulations.

The last module was the Adafruit RFM95W LoRa Radio Transceiver [49] (manufactured by Adafruit Industries, New York, NY, USA), capable of transmitting data over distances of several kilometers, and making it suitable for applications in IoT networks. The module offers a balance between performance and affordability, having a price of approximately 20 euros. For our research, we created a point-to-point network using the LoRa protocol.

A comparison of the modules is presented in Table 1, focusing on their relevant specifications.

To establish point-to-point communication and exchange messages between the onboard unit (OBU) and the RSU, the modules must be connected to a microcontroller, either directly or through adapters. Since we used test devices that work with low hardware resources, we selected commercial development boards that are based on microcontrollers. These boards are suitable because our modules do not need complex software and require only standard interfaces, such as UART or SPI. To connect these boards with the modules, we used a few adapter boards: prototyping shields for Bluetooth, nRF24, and LoRa modules, a commercial shield for XBee modules, and one for the GNSS receiver.

One of the development boards we used was the Arduino Mega 2560, a platform based on the Atmel ATmega2560 microcontroller that is manufactured by Arduino, Turin, Italy. We chose this board because it has four hardware serial ports for connecting our UART modules and the GNSS receiver, supports both 3.3 V and 5 V logic levels, and is compatible with standard Arduino shields. This compatibility made it easy to test our modules safely with minimal wiring. From the same manufacturer, we also used an Arduino Uno R3. We chose this board because its SPI pins can be connected directly to an external shield, eliminating the need for additional wiring required with the Arduino Mega.

The SparkFun XBee Shield [54] (manufactured by SparkFun Electronics, Niwot, CO, USA) that we chose is an adapter board designed to provide electrical connections between Digi XBee modules and the Arduino board. Since its operation is based on the virtual UART port on the D2 and D3 pins of the Arduino Uno and we wanted to use a hardware serial port, we slightly modified the shield to connect the module to an UART interface on the Mega board. Additionally, the shield addresses the power supply and level-shifting challenges of the XBee module, which operates at 3.3 V.

The last adapter board was the SparkFun GPS Shield (manufactured by SparkFun Electronics, Niwot, CO, USA), which was designed to provide the required connections between an Arduino board and a GNSS receiver [55]. It operates at a supply voltage between 3 and 4.2 V, and, as in the previous case, the shield provides the power supply voltage and manages the level-shifting of the data lines. The GNSS receiver type was EM-506N5 [56], manufactured by USGlobalSat, New Taipei City, Taiwan, having a 2.5 m horizontal position accuracy.

Two antenna types were used, one for the 2.4 GHz modules and another for the LoRa module. The 2.4 GHz antenna is an omnidirectional one, with a gain of 3.5 dBi, selected based on previous research by the authors [29]. For the 868 MHz spectrum, we used a tilt-swivel 1/2-wave whip antenna, recommended by the LoRa module supplier [57].

### 4.3. Message Exchange Protocol and Testing Procedure

**Figure 8:**
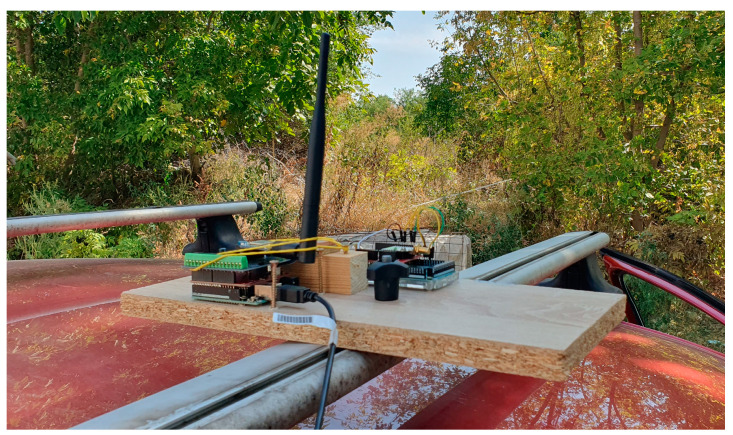
Onboard module placement.

The point-to-point communication setup between the RSU and the OBU was designed to evaluate the performance of the wireless communication modules under real-world conditions. For each test, a pair of identical communication modules was used. One module, connected to a microcontroller-based board, was placed on the roadside at approximately 1.5 m above the ground, while the other module was installed on top of a moving vehicle, also at approximately 1.5 m above the ground (Figure 8), acting as the OBU.

As the vehicle traveled along the road at approximately 50 km/h (the usual legal speed limit in cities), it entered the communication range of the RSU. Once a connection was established, the RSU began the message exchange by sending a fixed payload of 32 bytes of data to the OBU. The OBU, equipped with a GNSS device, received these data and immediately responded with a 32-byte message that included its current coordinates. The payload size was chosen based on the comparison in Table 1, ensuring that no module was required to send the payload in more than one packet. The communication protocol was configured so that if the receiving side did not get a response, it would wait for a 500 ms timeout before resending the message. This process was also set to continue as long as the vehicle remained within the communication range of the RSU. The goal was to capture as many successful message exchanges as possible before the vehicle went out of range. Five datasets (meaning five trips, from one end of the road to the other) were collected for each setup.

In addition to the number of successful message exchanges and the connection duration, which served as primary performance metrics, the inclusion of coordinates in the OBU’s response allowed for mapping the locations where communication was established and lost, as well as mapping how communication behaved at specific points along the route. This information was used to calculate the effective communication range of each module and helped assess how the modules performed in terms of coverage and reliability in a V2I scenario.

Overall, the following terms and metrics were considered:

- **Communication range (CR)** is the distance between the points where communication is established and then lost, their coordinates being obtained by the GNSS receiver, measured in meters.
- **Communication time (CT)** represents the time during which the communication link between the RSU and the vehicle is successfully maintained. It starts from the moment the vehicle enters the coverage area of the RSU and the first successful message exchange occurs and ends when the vehicle exits the communication range of the RSU and the connection is lost (i.e., when no further message exchanges can take place).
- **Successful message exchange (SME)** is defined by a communication that includes both sending a 32-byte message from the RSU and receiving a 32-byte response from the OBU. The number of successful message exchanges was counted.
- **Effective communication rate (ECR)** is obtained by combining the number of successful message exchanges with the total time the module maintains communication to help normalize the comparison between fast-exchange, short-range modules and slow-exchange, long-range modules. It is measured in successful message exchanges per second, over the communication range.
- **Timeout**—each time the RSU sends a 32-byte message to the OBU, it waits for a reply. If the reply is not received within the specified timeout period, it is counted as a timeout. The total number of timeouts was calculated.
- **Connection establish distance (CED)** is the distance between the RSU and the OBU moving toward it, at the moment of the first successful message exchange. As the vehicle approaches the RSU, the system continuously attempts to exchange messages. This distance was obtained by using the OBU coordinates supplied by the GNSS receiver (relative to the coordinates of the RSU), measured in meters.
- **Connection lost distance (CLD)** is the distance at which the communication between OBU and RSU is lost as the vehicle moves away. This is the point from which the system can no longer successfully exchange messages, either due to signal alteration, interference, or the vehicle moving beyond the effective communication range of the RSU.
- **Maximum connection establish distance (MaxCED), maximum connection lost distance (MaxCLD)**—the maximum values obtained from the 5 measurement sets in the urban environment and the 10 measurement sets in the inter-urban environment (5 from the open field and 5 from the forest). They were used to calculate the error rate, described next.
- **Error rate (ER)** is the percentage of unsuccessful communication attempts (timeouts) relative to the total number of attempts (successful message exchanges plus timeouts). The error rate is calculated at various distance intervals (every 10% of maximum CED or CLD) for each module and environment. This helps in understanding how the reliability of communication changes with distance and environmental conditions.
- **Operational communication range (OCR)** is the range where communication is technically possible, though not guaranteed to be entirely reliable. It was calculated by determining the maximum communication range at which the error rate never reached 100% for all measurements. While the determination of the OCR considers the extreme case where message exchange was impossible, the results presented in this paper can be used to determine a reliable communication range (RCR) at any error rate threshold suitable for specific application requirements.

## 5. Results

Based on the testing procedure previously presented, our experimental evaluation of the wireless communication modules aimed to provide valuable information regarding their performance in various environments and road types. In this section, we present the results of our tests conducted with each of the previously detailed modules in urban, open-field, and forest environments (the latter two were considered together as inter-urban roads). Comparing the performance of each module and highlighting the impact of environmental factors allowed us to interpret how each module operates in real-world conditions, thus providing support in selecting the appropriate communication technologies for integrating connected vehicles into IoT ecosystems.

Before presenting the evaluation results, we need to clarify some specific aspects of the measurement and data collection process:

- For the nRF24L01 module, due to the short communication times (ranging from less than a second up to 8 s), we were unable to obtain accurate GNSS positioning data for the two points needed to calculate the CED and CLD.
- For the Adafruit RFM95W LoRa module, in the forest environment, after the first test we concluded that the length of the road was insufficient. Since we could not find another suitable road section, no data were ultimately collected.

### 5.1. Communication Range

**Figure 9:**
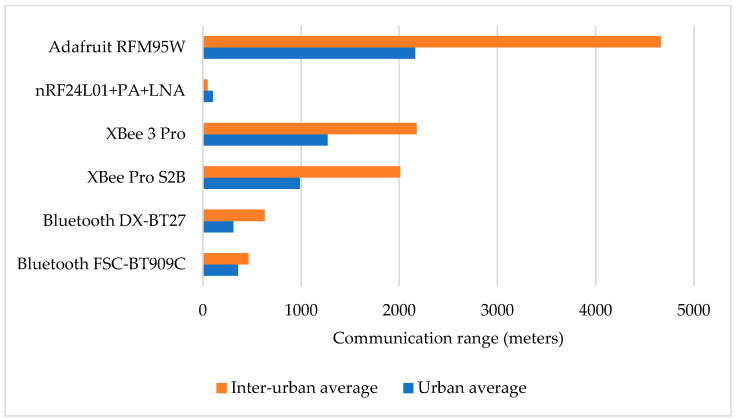
Communication ranges for different road types.

The communication range directly impacted the effectiveness of V2IoT communications in different environments. Figure 9 shows the average values collected for the two road types: urban and inter-urban.

Most modules showed substantial increases in the communication range in inter-urban environments, often doubling the values observed in urban settings. The LoRa module demonstrated the highest communication ranges for both road types (with more than 4 km on inter-urban roads), outperforming all other technologies. In contrast, the nRF24L01 consistently displayed the shortest communication ranges in all tests, contrary to the results of static tests previously presented in [36] and to the advertised specifications of the module. Its average range for the urban road type was approximately 103 m, which further decreased to about 48 m in inter-urban settings, likely due to a susceptibility to environmental factors affecting signal propagation. The Bluetooth modules had moderate communication ranges, with the DX-BT27 achieving an average of over 600 m on the inter-urban roads, which is impressive, considering that it is a PAN-dedicated technology. The XBee modules demonstrated high performances, with ranges exceeding 1000 m for the urban road type and over 2000 m for the inter-urban one, achieving a maximum communication range of 2739 m, closely matching the advertised specifications.

In addition to analyzing the communication range, it is important to consider the consistency of each module’s performance across different road types. Variability in communication ranges can significantly impact the reliability of V2IoT applications. In this regard, we examined the standard deviation of the communication ranges (see Table 2). In this table the last column is color-coded to provide a quick visual interpretation of variability in communication ranges. Green shades represent low standard deviations (below 10% of the mean), indicating consistent performance; yellow shades denote moderate variability (10–20%), and orange to red shades highlight high variability (above 20%), signaling significant inconsistency.

The calculated percentage based on the standard deviation revealed mostly moderate values and a significant variability in communication range consistency across different road types. The comparison between urban and inter-urban roads showed that inter-urban settings generally demonstrated greater variability in the communication range, particularly for modules such as the nRF24 and Bluetooth FSC-BT909C. This higher variability suggests that open fields create more challenging conditions for maintaining stable communication. In contrast, urban roads offer a more controlled setting with numerous fixed obstacles and structures, where most modules experienced low to moderate deviations and fewer disruptions to range stability. The RFM95W LoRa module stood out due to its low variability across both settings, showing robustness in diverse conditions. Additionally, the Bluetooth DX-BT27 module demonstrated consistency with similar standard deviation values across both road types, while the XBee 3 Pro showed improved performance compared to the older XBee Pro S2B module, reflecting its enhanced design and stability.

### 5.2. Effective Communication Rate

**Figure 10:**
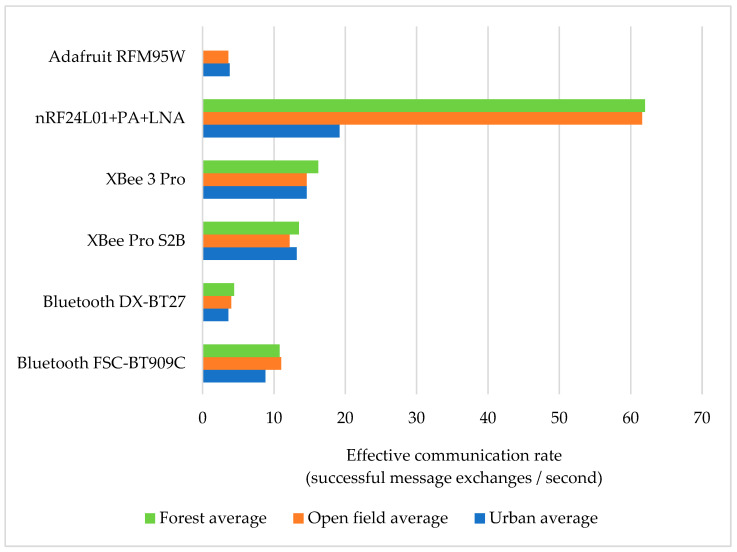
Effective communication rate for different environments.

While measuring how far the modules can exchange messages is important, it is not always relevant for assessing a module’s performance, as some may have shorter ranges but compensate with greater data rates. In this regard, we calculated the ECR, thus being able to identify which solution offered the optimal balance between range and message exchange efficiency. The results of this analysis are presented in Figure 10.

The nRF24L01 technology proved to have the highest effective communication rate, especially on the inter-urban roads, providing way faster message exchanges than the other modules, while LoRa offered long-range communication with slower data exchange (as expected). XBee modules provided a stable and high communication rate and a notable consistency across all road types. Bluetooth modules maintained stable performance, offering moderate rates across environments, but without excelling in any specific area.

### 5.3. Distances from the Roadside Unit

**Figure 11:**
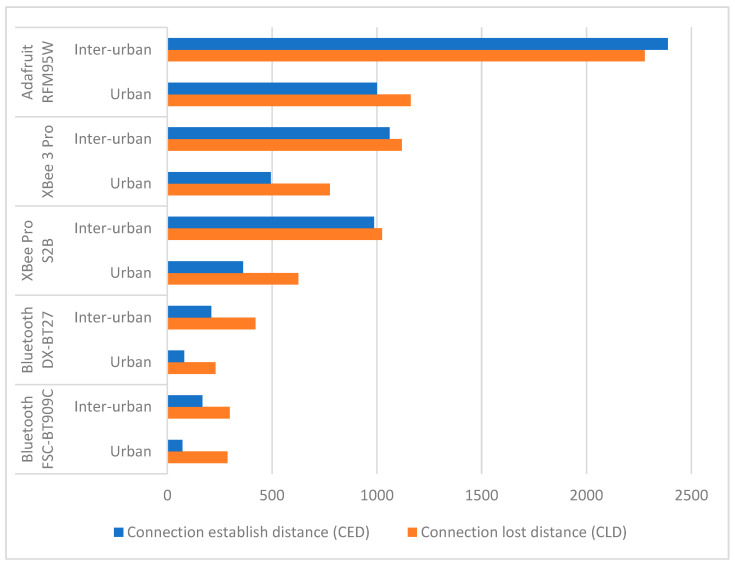
Average values of the CED and CLD for different road types.

During the tests, we noticed that the two distances, the RSU location to the point of connection (CED) and the RSU location to the point of disconnection (CLD), did not always have values close to each other (in one set of measurements). Sometimes, the difference was significant, making their evaluation worthwhile. A comparison of the two distances for different road types is presented in Figure 11.

In most cases, the CED was shorter than the CLD. This can be attributed to the processes involved in establishing a connection, such as synchronization, authentication, or the exchange of control information, which usually require a higher signal quality (influenced by environmental factors, such as trees, buildings, or other vehicles) and take time to complete before data transmission can begin. The protocols and hardware characteristics of each technology influence how initial connections are established and maintained. For example, Bluetooth devices require a pairing process involving device discovery and authentication. This, combined with the significant impact of environmental factors, results in a much shorter CED compared to other technologies. ZigBee devices are also affected because they must join a network by discovering and associating with a coordinator, which involves exchanging network and security parameters. LoRa, on the other hand, seems to be less affected, most likely because it usually operates in a connectionless manner.

### 5.4. The Error Rate

One aspect that remains to be evaluated is the message exchange interruption, specifically called “timeout”. As mentioned above, a timeout occurs if the RSU does not receive a reply within a specified timeframe, indicating a disruption or degradation in the communication link. A high number of timeouts suggests that the message exchange may not be dependable in certain environments or over specific distances. The impact of timeouts is determined by the error rate, previously defined, for every module, across different environments (urban, open field, and forest) at incremental percentages of MaxCED and MaxCLD, in order to identify the reliable communication ranges (distances where the error rate remains under an acceptable threshold). Table 3, Table 4 and Table 5 show the average error rate values and Table 6 and Table 7 the maximum values. To quickly identify performances across modules and environments, these tables are color-coded using a red-yellow-green heat map. Red shades indicate high error rates, signaling poor communication reliability, yellow represents moderate error rates, and green highlights low error rates, reflecting more reliable communication.

For both Bluetooth modules, error rates were high at longer distances (for distances over 70% of the MaxCED or MaxCLD, the timeouts accounted for more than 70% of the total communication attempts), and they had better performance at short distances (0–30% of the MaxCED or MaxCLD). The comparison between MaxCED and MaxCLD revealed that error rates were slightly lower for the latter, because maintaining an existing connection is generally more robust over longer distances than establishing a new one.

The Bluetooth FSC-BT909C module showed higher error rates in open-field environments compared to urban or forest settings. This could be attributed to a combination of factors related to the following:

- antenna design,
- susceptibility to multipath reflections, as in urban and forest environments it can sometimes enhance signal strength through constructive interference,
- receiver sensitivity, because the FSC-BT909C has a lower receiver sensitivity (−86 dBm [50]) compared to the DX-BT27 (−95 dBm [51]), making it less capable of detecting weaker signals prevalent in open fields,
- electromagnetic interference in open fields,
- quality of RF components,
- protocol error correction methods, and so on.

Both XBee modules had higher error rates at longer distances (80%–100%), especially in urban and forest environments. However, XBee 3 Pro outperformed XBee Pro S2B at mid to short distances, achieving significantly lower error rates and reaching 0% quicker. XBee Pro S2B may be preferable for maintaining connections at longer distances, especially in urban environments, even with the risk of a higher error rate (more than 50%, but not reaching 100%).

Compared to Bluetooth modules, XBee modules consistently outperformed them across various environments and distances. Bluetooth modules tended to have higher error rates, especially at distances beyond 50% of their MaxCED and MaxCLD. In contrast, XBee modules maintained more reliable connections over longer distances, with error rates decreasing rapidly as the distance decreased.

Regarding the nRF24L01 module, error rates were 0% across all intervals for both the CED and CLD, suggesting consistently reliable performance at the tested ranges.

For the LoRa module, error rates increased significantly at distances beyond 60% of their MaxCED and MaxCLD, with generally higher values in the urban environments compared to the open field. Compared to Bluetooth and XBee modules, the LoRa module exhibited error rates with similar values or slightly higher ones, especially for distances higher than 30% of the MaxCED and MaxCLD, and despite the high error rates at maximum distances, it maintained connections over longer ranges than Bluetooth and XBee modules.

Using average error rate values provides a general idea of the typical performance of the modules, as this approach reduces the impact of extreme values or data anomalies. This can be helpful when designing systems where the impossibility of sending a message is occasionally acceptable but is less useful for critical applications. In this regard, Table 5 and Table 6 present the maximum error rates observed for each distance interval across different road types and measurement sets, enabling the determination of the OCR, where the error rate never reached 100%.

As seen before, the nRF24L01 module had excellent performance, with a 0% error rate across all distance intervals, indicating highly reliable communication within its limited communication range. The XBee 3 Pro outperformed the other modules at mid-distances in establishing and maintaining connections. The LoRa module demonstrated higher error rates compared to other modules, across all distance intervals, indicating challenges in maintaining connections in urban environments, especially at longer distances. For the XBee and Bluetooth modules, the probability of message exchange failure (100% error rate) was higher when the vehicle was approaching the RSU (within the MaxCED), with the modules exhibiting difficulties in establishing new connections and successfully initiating message transmission.

For the inter-urban environment, the comparison between the MaxCED and MaxCLD error rates showed that the values were very similar across most of the intervals. This is a notable difference compared to the urban environment, where the MaxCED had more intervals with a 100% error rate than the MaxCLD, indicating similar challenges in both establishing and maintaining connections at longer distances. One possible reason is that the signal strength decreased more rapidly over a distance, and, for the inter-urban environment, MaxCED and MaxCLD were higher compared to the urban setting. However, there was an exception: the LoRa modules performed slightly better in the inter-urban environment, considering that lower frequencies (868 MHz) have better propagation characteristics, including less path loss, diffraction, and better penetration through obstacles compared to higher frequencies (2.4 GHz).

### 5.5. Operational Communication Range

**Figure 12:**
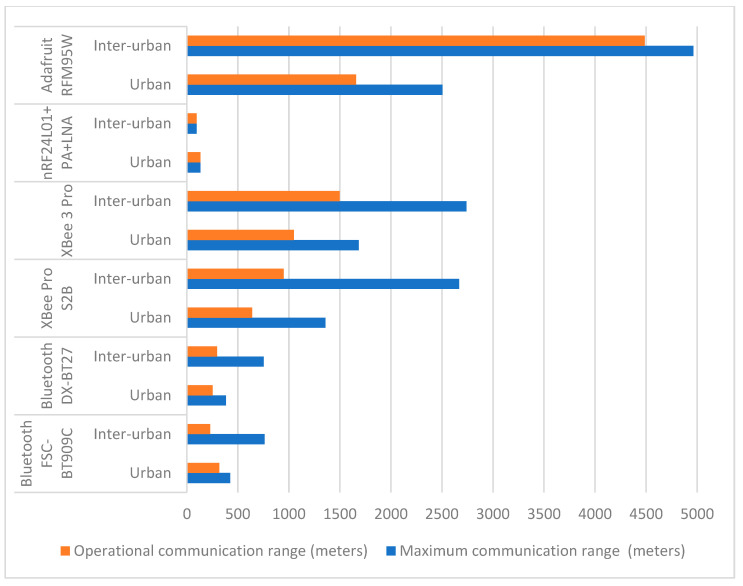
Maximum communication range vs. operational communication range for different road types.

Based on the results presented in Table 5 and Table 6, we calculated the OCR for each module and road type, excluding cases where the error rate reached 100%. Figure 12 presents these results.

The OCR varied for different modules and environments, reflecting each device’s ability to maintain reliable communication under worst-case conditions. All modules had smaller OCRs than their maximum measured values, except for the nRF24L01. However, while the nRF24L01 maintained full reliable communication within its operational range without reaching a 100% error rate, its range was significantly limited compared to other modules. Bluetooth performed better on the urban road type, with OCRs approaching their maximum range (approximately 70%), while showing marginal or no increase for the inter-urban roads. XBee modules provided moderate to long OCRs but experienced significant increases in the error rate beyond certain distances (40–50% of their maximum range), limiting their effectiveness for very long-range communications. LoRa demonstrated the best performance in avoiding 100% error rates over long distances, having an OCR at 66% of its maximum range on the urban road type and at 90% on the inter-urban ones.

## 6. Discussion

The aim of this research was to assess the performance of multiple LPLR low-cost communication technologies and modules, and to understand their strengths and weaknesses for both urban and inter-urban road-type applications, considering their integration in V2IoT solutions. In this regard, six modules were tested in three different environments (urban, open field, and forest) using technologies such as Bluetooth, ZigBee, nRF24, and LoRa, and analyzing metrics such as the maximum communication range, operational communication range, error rate, and effective communication rate.

In the urban environment, Bluetooth modules demonstrated limited OCRs (several hundred meters), generally experiencing higher error rates at longer communication distances. For the inter-urban road types, Bluetooth modules’ performance was further degraded (for the FSC-BT909C) or had minor improvements (DX-BT27), with an important reduction of the OCR compared to more than 700 m of its maximum communication range. The modules did not excel in ECR either, with only LoRa showing lower values. Another disadvantage is the initial handshake, which introduces higher latency and uncertainty. This often leads to a significant difference between the CED and CLD, resulting in shorter distances between the OBU and the RSU when actual message exchange begins. Considering these aspects, Bluetooth technology seems more suitable for short-range, high-data-rate applications, especially in urban environments, and for those where occasional message failures are acceptable (non-critical messaging). It is less suitable for critical applications demanding high reliability over longer distances. This technology is cost-effective and offers viable options for IoT networks in controlled environments, such as smart homes, parking management, localized vehicle diagnostics, toll payment systems, or personal IoT ecosystems, particularly due to its simplicity, compatibility with common devices, and widespread integration in vehicles. However, their short range may limit their effectiveness for broader public infrastructure deployment, where long-range communication is essential.

The XBee Pro S2B and XBee 3 Pro modules demonstrated better performance than Bluetooth, offering extended ranges and more consistent performance across all environments, although error rates increased notably at longer distances. They are well suited for medium- to long-range communication needs, where reliability is important but also not critical, offering a balance between OCR (particularly the XBee 3 Pro, with 1.5 km in the inter-urban environment) and ECR. While XBee modules offer increased range compared to Bluetooth, they can still be affected by longer times needed to establish a new connection for a mobile device and shorter initial connection distances due to handshake delays and connection stability. However, once the connection is established, it remains robust, even as the vehicle moves farther away.

For urban environments, the XBee modules offer scalability potential and higher ECR, making them ideal for mid-range V2IoT applications, where long-distance communication is less critical (an average communication range of about 1 km is typically sufficient for most applications). Mesh network configurations offer additional flexibility, providing redundancy and extending the network’s range. This can be beneficial in urban environments if long-range communication is needed and continuous power is more readily available for routers.

For the inter-urban road types, both modules demonstrated impressive communication ranges, with the XBee 3 Pro showing a maximum communication range of 2.7 km, while the XBee Pro S2B reached 2.6 km. However, the XBee Pro S2B exhibited higher error rates compared to the XBee 3 Pro, indicating that the newer XBee 3 Pro might be more resilient in maintaining communication in open, long-range environments.

In public use, XBee may be integrated into applications where vehicles interact with smart parking systems, traffic lights, automated toll collection, or other infrastructure elements to improve traffic flow and safety. In private V2IoT systems, XBee modules can handle real-time data from multiple vehicles on a site, enabling seamless integration into broader industrial automation systems. Vehicles may also be integrated into smart home systems, where cars are enabled to communicate with the home automation network, with ZigBee networks being a popular choice for home devices.

The nRF24L01 module consistently displayed the shortest communication ranges among all tested modules (around 100 m) and exhibited high variability in communication range across inter-urban tests. However, it showed small variations in performance across different environments, maintaining short reliable communication distances with 0% error rates and achieving the highest ECR (at least four times more message exchanges per second than the other modules), thus being ideal for high-frequency, low-latency applications that prioritize fast message exchanges in short ranges, potentially suitable for critical safety messaging.

As previously mentioned, we were unable to determine the CED and CLD for the nRF24L01 module due to its short communication times. However, it is worth noting that message exchanges began when the vehicle was very close to the RSU.

In summary, it can be noted that the nRF24L01 technology performed best at short distances, with high messaging rates and no errors. This makes it suitable for highly localized cost-effective applications, such as small industrial setups where devices are typically located in close proximity. Vehicle owners can integrate their cars into private IoT networks for security and/or monitoring purposes. This could include real-time tracking of vehicle location, monitoring for unauthorized access, remote diagnostics, or maintenance alerts. In private communities, vehicles can interact with the community’s IoT network, enabling gate access control, automated security checks, or communication with community services. However, its limited range constrains its use in broader V2IoT deployments.

The Adafruit RFM95W LoRa module demonstrated the highest performance compared to the other modules, particularly for long-range communication across both urban and inter-urban road types. In our tests, the LoRa module consistently showed the highest maximum communication range across all environments, with values exceeding 2.5 km on the urban road type and reaching nearly 5 km on the inter-urban one, this being a significant advantage over the other technologies.

Regarding the OCR, LoRa achieved about 1.7 km on the urban road and nearly 4.5 km on the inter-urban one, also considerably higher than any other tested module. Despite its lower ECR, the long-range reliability and the lower average error rates at extended distances make it ideal for applications requiring long communication distances and low-frequency message exchanges. Indeed, the lower data rates are a limitation, but this can be mitigated if the data payloads are small, which is often the case in many IoT applications.

In urban areas, the module maintained a stable connection with fewer instances of 100% error rates compared to Bluetooth and XBee modules, making it highly suitable for critical applications requiring reliable long-range communication. On the inter-urban roads, where other technologies might struggle with maintaining a connection due to signal alteration or interference from environmental factors, the LoRa module also performed robustly. Although it showed some slight increases in error rates at longer distances, it still outperformed Bluetooth and XBee, proving LoRa’s efficiency in rural or remote V2IoT scenarios. The ability to maintain a consistent connection over several kilometers on both urban and inter-urban road types suggests that LoRa can be deployed in a wide network of sensors, vehicles, and roadside infrastructures without requiring frequent gateway deployment.

Another important advantage of LoRa in V2IoT applications is its low current consumption, which makes it especially useful for vehicles that may need to maintain communication over long distances without drawing excessive power from their batteries. This is required in applications such as vehicle fleet management, where vehicles need to communicate with central systems over large geographical areas, often in areas with limited infrastructure. Additionally, LoRa networks are relatively cost-effective to deploy, especially when compared to traditional cellular-based V2I systems, which involve higher infrastructure costs and maintenance requirements.

For V2IoT applications, LoRa can be highly beneficial in scenarios where vehicles need to communicate with RSUs or infrastructure in remote areas. Vehicles equipped with LoRa modules could act as mobile environmental sensors, transmitting air quality, temperature, or traffic data over long distances to a central monitoring system. Additionally, LoRa can be used in smart agriculture, where vehicles in farming areas need to communicate with sensors or equipment over large fields, to monitor field conditions, automate planting and irrigation, or track livestock. For private companies with their own vehicle fleets, LoRa can be used to effectively track vehicles over long distances on urban or inter-urban road types without requiring high data rates. However, LoRa’s low data rate may limit its use in applications that require real-time communication or high-bandwidth data transfer, making it more suitable for periodic updates rather than continuous data streams.

## 7. Conclusions

The primary goal of this research was to evaluate the performance of six LPLR and low-cost communication modules across different road environments, focusing on their potential integration into V2IoT solutions. By testing these modules in urban, open-field, and forest environments, we were able to evaluate how technologies, such as Bluetooth, ZigBee, nRF24, and LoRa, performed in terms of range, communication rates, or error rates.

While each communication technology tested proved its strengths and limitations, LoRa seemed to be the best choice for long-range, low-power V2IoT applications, across all environment types, where other technologies may fail due to increased error rates and reduced operational communication ranges. XBee modules offered a balanced solution for medium-range applications, where reliability and scalability are important, while Bluetooth and nRF24 were better suited for short-range, high-data-rate scenarios in urban environments.

Future work will focus on expanding the test scenarios to cover more varied and complex real-world conditions. This includes testing the performance of communication modules in various weather conditions (rain, fog, snow, or extreme temperatures), in environments with hilly terrain or curved roads, as well as under high-speed situations, which are common on highways.

In addition, future research will investigate the performance of these technologies in multi-hop or mesh communication scenarios, which could potentially help extend the reliable range of short-range modules, such as Bluetooth or nRF24. Testing multiple RSUs and OBUs, as well as evaluating handovers between RSUs, will be considered to properly understand how vehicles move through a network of roadside infrastructure and help assess the scalability and resilience of V2IoT networks.

An important research area will be the adaptation of these technologies to road environments, by developing standardized V2IoT protocols that enable vehicles, RSUs, and infrastructure to connect and communicate efficiently, reliably, and safely.

Lastly, the creation of cooperative, opportunistic communication environments using the investigated technologies could help in improving the reliability and resilience of Internet of Vehicles and V2IoT applications, facilitating their expansion in the future.

## Figures and Tables

**Table 1:** Specifications of the communication modules.

Module Type		FSC-BT909C [50]	DX-BT27 [51]	XBee Pro S2B [46]	XBee 3 Pro [47]	nRF24L01 +PA+LNA [48,52]	Adafruit RFM95W [53]
**Communication Protocol**		Bluetooth Classic BR-EDR	Bluetooth Low Energy (BLE v5.1)	ZigBee Pro	ZigBee 3.0	Proprietary RF protocol	LoRa
**Operating Frequency**		2.4 GHz	2.4 GHz	2.4 GHz	2.4 GHz	2.4 GHz	868 MHz
**Transmit Power**		18.5 dBm	Up to 13.5 dBm	18 dBm	19 dBm	20 dBm	20 dBm
**Range**		420 m	580 m	Up to 3200 m	Up to 3200 m	Up to 1100 m	Approx. 2 km
**Modem** **Current** **Consumption**	**RX**	44 mA	10 mA	47 mA	17 mA	45 mA	12.1 mA
	**TX**	44 mA	10 mA	117 mA	140 mA	115 mA	120 mA
	**Lowest**	1.5 mA	0.25 mA	3.5 μA	2 μA	0.9 μA	0.2 μA
**Max Payload**		128 bytes	253 bytes	84 bytes	84 bytes	32 bytes	64 bytes
**Interface**		UART	UART	UART	UART	SPI	SPI

**Table 2:** Communication range standard deviation.

Module	Environment	Average Communication Range	Communication Range Standard Deviation	Percentage from the Average Communication Range
Bluetooth FSC-BT909C	Urban	357.66 m	52.27 m	14.61%
	Inter-urban	464.60 m	175.04 m	37.68%
Bluetooth DX-BT27	Urban	310.53 m	40.57 m	13.07%
	Inter-urban	630.42 m	93.76 m	14.87%
XBee Pro S2B	Urban	986.65 m	284.60 m	28.85%
	Inter-urban	2010.51 m	431.13 m	21.44%
XBee 3 Pro	Urban	1270.30 m	269.33 m	21.20%
	Inter-urban	2178.36 m	298.09 m	13.68%
nRF24L01+PA+LNA	Urban	103.40 m	20.14 m	19.48%
	Inter-urban	47.66 m	36.32 m	76.20%
Adafruit RFM95W	Urban	2162.05 m	223.60 m	10.34%
	Inter-urban	4665.22 m	270.94 m	5.81%

**Table 3:** Error rate average values for the Bluetooth modules.

			Bluetooth FSC-BT909C				Bluetooth DX-BT27		
			Urban	Open Field	Forest		Urban	Open Field	Forest
Error rate average values (in %) for	90–100%	of the MaxCED	82	100	89	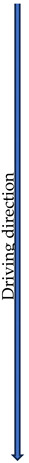	80	80	100
	80–90%		80	100	82		80	80	100
	70–80%		80	100	72		84	80	88
	60–70%		80	81	23		57	67	69
	50–60%		80	67	3		40	60	39
	40–50%		62	60	0		33	48	14
	30–40%		46	26	0		5	40	2
	20–30%		33	16	0		0	19	0
	10–20%		25	0	1		0	0	0
	0–10%		1	0	0		0	0	0
Error rate average values (in %) for	0–10%	of the MaxCLD	0	0	0		0	0	0
	10–20%		0	0	0		0	2	0
	20–30%		0	6	0		8	7	0
	30–40%		0	49	0		12	43	1
	40–50%		0	74	0		15	53	24
	50–60%		8	97	0		56	58	42
	60–70%		40	100	0		61	62	78
	70–80%		71	100	24		66	71	81
	80–90%		80	100	91		77	72	95
	90–100%		80	100	92		81	78	100

**Table 4:** Error rate average values for the XBee modules.

			XBee Pro S2B				XBee 3 Pro		
			Urban	Open Field	Forest		Urban	Open Field	Forest
Error rate average values (in %) for	90–100%	of the MaxCED	69	67	86	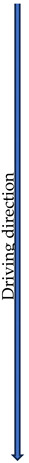	80	83	99
	80–90%		62	59	54		89	46	23
	70–80%		69	79	52		6	44	1
	60–70%		60	38	50		0	26	2
	50–60%		46	40	4		0	1	0
	40–50%		40	8	6		0	0	0
	30–40%		41	0	25		0	0	0
	20–30%		36	2	25		0	0	0
	10–20%		2	1	2		0	0	0
	0–10%		0	0	0		0	0	0
Error rate average values (in %) for	0–10%	of the MaxCLD	0	0	0		0	0	0
	10–20%		0	0	0		0	0	0
	20–30%		0	0	0		0	0	0
	30–40%		2	0	1		0	0	0
	40–50%		2	7	34		0	0	2
	50–60%		4	16	84		6	0	59
	60–70%		28	19	100		71	0	88
	70–80%		43	20	100		100	8	99
	80–90%		50	47	100		100	41	100
	90–100%		67	86	100		100	70	100

**Table 5:** Error rate average values for the nRF24L01 and LoRa modules.

			nRF24L01+PA+LNA				Adafruit RFM95W LoRa		
			Urban	Open Field	Forest		Urban	Open Field	Forest
Error rate average values (in %) for	90–100%	of the MaxCED	0	0	0	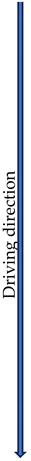	80	96	-
	80–90%		0	0	0		83	92	-
	70–80%		0	0	0		60	65	-
	60–70%		0	0	0		40	47	-
	50–60%		0	0	0		6	28	-
	40–50%		0	0	0		2	15	-
	30–40%		0	0	0		2	7	-
	20–30%		0	0	0		0	7	-
	10–20%		0	0	0		1	4	-
	0–10%		0	0	0		0	1	-
Error rate average values (in %) for	0–10%	of the MaxCLD	0	0	0		1	1	-
	10–20%		0	0	0		3	3	-
	20–30%		0	0	0		14	6	-
	30–40%		0	0	0		29	14	-
	40–50%		0	0	0		52	17	-
	50–60%		0	0	0		69	24	-
	60–70%		0	0	0		83	27	-
	70–80%		0	0	0		91	45	-
	80–90%		0	0	0		98	64	-
	90–100%		0	0	0		100	86	-

**Table 6:** Error rate maximum values in an urban environment.

Urban Environment			Bluetooth FSC-BT909C	Bluetooth DX-BT27	XBee Pro S2B	XBee 3 Pro	nRF24L01 +PA+LNA	Adafruit RFM95W LoRa	
Error rate maximum values (in %) for	90–100%	of the MaxCED	100	100	100	100	0	100	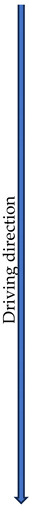
	80–90%		100	100	100	100	0	100	
	70–80%		100	100	100	100	0	100	
	60–70%		100	100	100	100	0	93	
	50–60%		100	100	100	100	0	14	
	40–50%		100	100	100	3	0	4	
	30–40%		100	25	100	0	0	5	
	20–30%		100	0	100	0	0	2	
	10–20%		67	0	11	0	0	2	
	0–10%		7	0	0	0	0	2	
Error rate maximum values (in %) for	0–10%	of the MaxCLD	0	0	0	0	0	3	
	10–20%		0	0	0	0	0	14	
	20–30%		0	40	0	0	0	61	
	30–40%		0	40	6	0	0	84	
	40–50%		0	60	11	46	0	91	
	50–60%		27	75	14	53	0	92	
	60–70%		71	75	64	92	0	100	
	70–80%		83	80	100	100	0	100	
	80–90%		100	100	100	100	0	100	
	90–100%		100	100	100	100	0	100	

**Table 7:** Error rate maximum values in an inter-urban environment.

Inter-Urban Environment			Bluetooth FSC-BT909C	Bluetooth DX-BT27	XBee Pro S2B	XBee 3 Pro	nRF24L01 +PA+LNA	Adafruit RFM95W LoRa	
Error rate maximum values (in %) for	90–100%	of the MaxCED	100	100	100	100	0	100	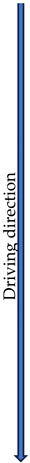
	80–90%		100	100	100	100	0	100	
	70–80%		100	100	100	100	0	87	
	60–70%		100	100	100	100	0	95	
	50–60%		100	100	100	4	0	45	
	40–50%		100	100	26	0	0	22	
	30–40%		100	100	100	0	0	13	
	20–30%		80	50	100	0	0	15	
	10–20%		4	0	5	0	0	9	
	0–10%		0	0	2	0	0	3	
Error rate maximum values (in %) for	0–10%	of the MaxCLD	0	0	0	0	0	4	
	10–20%		0	6	0	0	0	7	
	20–30%		20	25	0	0	0	14	
	30–40%		100	100	3	0	0	27	
	40–50%		100	100	100	6	0	34	
	50–60%		100	100	100	100	0	61	
	60–70%		100	100	100	100	0	87	
	70–80%		100	100	100	100	0	87	
	80–90%		100	100	100	100	0	90	
	90–100%		100	100	100	100	0	100	

## Data Availability

The datasets presented in this article are not readily available because the data are part of an ongoing study. Requests to access the datasets should be directed to Valentin Iordache.
